# Reconstruction of Oronasal Fistula with Tongue Flap: A Cleft Palate Report

**DOI:** 10.3390/bioengineering9090455

**Published:** 2022-09-08

**Authors:** Francisco Vale, Flávia Pereira, José Saraiva, Eunice Carrilho, Madalena Prata Ribeiro, Filipa Marques, Raquel Travassos, Catarina Nunes, Anabela Baptista Paula, Inês Francisco

**Affiliations:** 1 Faculty of Medicine, Institute of Orthodontics, University of Coimbra, 3004-531 Coimbra, Portugal; 2 Faculty of Medicine, Area of Environment Genetics and Oncobiology (CIMAGO), Coimbra Institute for Clinical and Biomedical Research (iCBR), University of Coimbra, 3004-531 Coimbra, Portugal; 3 Faculty of Medicine, Institute of Integrated Clinical Practice, University of Coimbra, 3004-531 Coimbra, Portugal; 4 Centre for Innovative Biomedicine and Biotechnology (CIBB), University of Coimbra, 3004-531 Coimbra, Portugal; 5 Clinical Academic Center of Coimbra (CACC), 3004-531 Coimbra, Portugal

**Keywords:** aesthetics, dental restoration, cleft palate, bone transplantation, oronasal fistula, orthodontics

## Abstract

Oronasal fistula can persist after conventional secondary alveolar bone graft surgery, which may lead to functional issues, such as regurgitation of fluids from the oral to the nasal cavity. This manuscript describes a clinical case of a patient with a bilateral cleft lip and palate that underwent tongue graft surgery for closure of an oronasal fistula after three failed local mucosa flap surgeries. The multidisciplinary treatment was comprised of orthodontic treatment, mucosa and alveolar grafts for palate closure and aesthetic rehabilitation of the anterior maxillary teeth. Smile aesthetics were noticeably improved, enhancing the patient’s self-perception and confidence.

## 1. Introduction

One of the most common congenital craniofacial deformities is cleft lip and palate (CLP), which affect 1 in 700 live births. The etiology of patients with CLP is multifactorial and still in study, but several genetic and environmental factors are already recognized as nefarious, such as viral infections, medication (e.g., anticonvulsants), drug use, smoking, and alcohol consumption during pregnancy. In early pregnancy, some nutritional deficits, such as folate deficiency, can also increase the risk of having a child with CLP [1].

Around the ages of 8 to 9, patients with CLP should undergo a secondary alveolar bone graft surgery before the eruption of canines or lateral maxillary incisors. This intervention will create bone support for the canine teeth, which may improve bone density maintenance in the grafted region. The bone graft will also provide enhanced support to the alar bases which in turn promotes nasal and lip symmetry, closure of the oronasal fistula, and cleft maxillary segment stabilization [2]. If, for any reason, this procedure fails, an oronasal fistula may persist due to a soft and hard tissue defect. The fistula will result in oronasal communication, which in turn can lead to abnormal speech, malocclusion, regurgitation of fluids from the oral to nasal cavities, deafness, severe facial deformity, and psychological impediments [3,4,5].

The local flap procedure, whilst important, may alone not be the most suitable technique to attempt full fistula closure. Subsequent mucoperiosteum scarring will create tension on the local flaps, which can lead to necrosis and palate cleft dehiscence [6,7]. Clinical characteristics such as the presence of large defects, local scar tissue due to previous repair attempts with local mucosa flap, difficult access, and location can contribute to the unpredictability of the local flap during treatment [3,7,8,9]. When these components are observed on the local flap, regional flaps (e.g., nasolabial or tongue) may be used in its stead [7,10].

This manuscript presents a clinical case of a patient with bilateral cleft lip and palate that underwent a tongue graft for oronasal fistula closure procedure after three failed local mucosa flap surgeries.

## 2. Materials and Methods

### 2.1. Diagnosis and Etiology

An 18-year-old male presented at the Institute of Orthodontics with a complete bilateral cleft lip and palate. The patient’s chief complaint was oronasal communication persistence after several surgeries. The patient also reported being dissatisfied with his smile and had low self-esteem. The patient’s medical history included a bilateral lip closure surgery at 2 months old, palate closure surgery at 8 years old, and three failed attempts of soft palate closure between the ages of 10 and 14. The patient was healthy and presented with no other medical conditions or syndromes. In terms of dental history, the patient had only undergone standard preventive procedures, such as pit and fissure sealants and annual scalings.

**Figure 1:**
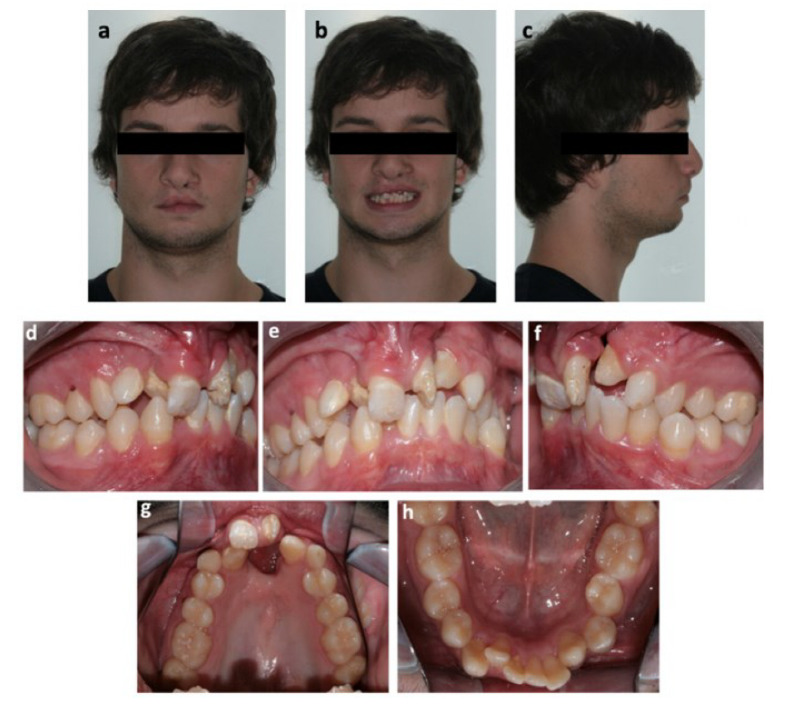
Pretreatment photographs: extraoral (**a**) frontal view; (**b**) frontal view with smile; (**c**) profile and intraoral (**d**) right side; (**e**) frontal; (**f**) left side; (**g**) superior arch; (**h**) inferior arch.

Extraorally, the patient presented with a convex profile and a flat nasal tip. From a fontal perspective, the patient’s face seemed symmetrical, and an increase of the nasal base width along with the presence of mild bilateral vertical scars on the upper lip due to the lip closure surgery were also observed. Additionally, the patient had a mesofacial pattern and an increased nasolabial angle as well as upper and lower lip retrusion. The mandibular dental midline was shifted 2 mm to the left and the maxillary dental midline 2 mm to the right in relation to the facial midline (Figure 1).

The functional examination showed that the temporomandibular joint was asymptomatic with no functional mandibular shift and deglutition was normal.

After an intraoral clinical evaluation, it was possible to observe a bilateral cleft of the primary palate, protruded premaxilla, maxillary lip curl with low insertion, hypomineralization of both maxillary central incisors and permanent superior canines, and adequate oral hygiene. The left central incisor showed an abnormal conical shape, and both central incisors were retroclined. The fistula dimensions were 12.0 × 13.0 mm (transversal × anteroposterior dimensions), and there was scar tissue surrounding the edges of the fistula (Figure 1). In terms of dental relations, on the right side the patient presented a molar Angle Class I, on the left side a molar Angle Class II and a bilateral canine Class II due to agenesis of the lateral maxillary incisors.

**Figure 2:**
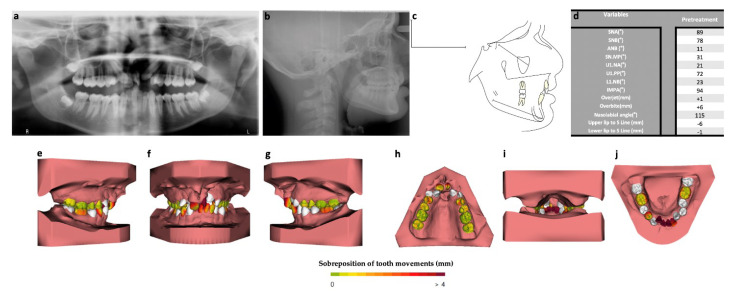
Pretreatment records: (**a**) orthopantomography; (**b**) lateral cephalogram; (**c**) cephalometric analysis; (**d**) dental casts; (**e**) right side; (**f**) frontal side; (**g**) left side; (**h**) superior arch; (**i**) back side; (**j**) inferior arch.

Radiographically, with the aid of orthopantomography, it was possible to confirm the agenesis of the upper laterals, but it was also possible to observe the retention of the primary maxillary canines, impaction of the four third molars, and severe root reabsorption of the 11 and 21 incisors (Figure 2).

The maxillary right canine and maxillary first left premolar were in a crossbite relationship. The maxillary arch was a V-shaped and the mandibular arch form was ovoid. The transpalatal arch width at the first molar was 39.5 mm on the maxilla and 42.0 mm on the mandible (−2.5 mm difference). The canine transpalatal arch width was 16.0 mm on the maxilla (reduced due to the agenesis of the lateral maxillary incisors) and 23.0 mm on the mandible (−7.0 mm difference). The patient had a normal overjet (+1.5 mm) but an increased overbite (+6.5 mm) (Figure 2). In addition, the patient presented a dental–maxillary discrepancy of 10.5 mm on the upper arch and 9.5 mm on the lower arch.

Lateral cephalogram analysis confirmed the presence of a skeletal Class II (ANB-11°) with mandibular retrusion (SNB−78°) (Figure 2). The maxillary incisors were retroclined (U1.PP−72°), and the mandibular incisors were slightly proinclined (IMPA−94°).

### 2.2. Treatment Objectives

The treatment objectives were: (1) correct the transverse maxillary deficiency; (2) restore normal maxillary arch form to allow for flap surgery and palate closure; (3) solve the mandibular crowding by extracting the first mandibular premolars; (4) create adequate spaces for prosthetic rehabilitation of the maxillary incisors, canines, and first premolars; (5) correct the dental midline deviations; (6) improve the overbite; (7) make room for a bone graft; (8) obtain functional protrusion and right and left laterality; and (9) long-term stability.

The multidisciplinary treatment was comprised of orthodontic treatment, mucosa and alveolar grafts for palate closure, and aesthetic rehabilitation of the anterior maxillary teeth.

### 2.3. Treatment Alternatives

Surgical closure of cleft palate is a necessary procedure, improving the phonation, articulation, and nasal and lip symmetry, which in turn improves the patient’s well-being and aesthetics [11]. Bone graft failure can occur when the cleft is notably large or when the mucoperiosteum flap inadequately covers the graft due to the formation of an extensive fibrous zone [12]. In this case report, several fibrous areas were observed, and these may have been the result of the multiple surgeries that had been performed on the patient between the ages of 10 to 14.

As an alternative to bone graft surgery, the osteogenic distraction technique can be used to improve alveolar bone. This procedure facilitates mucous coverage and new bone formation, reducing the size of the cleft palate [13]. The osteogenic distraction protocol offers several advantages, one of them is the lack of need for a second intervention site since there is no graft, this technique also allows for an expansion of the dental arch, making it suitable for crowding cases whilst simultaneously improving the morphology of the nasal septum and maxilla. However, this procedure requires several adjustments during distraction and a second surgical intervention to remove the distractor [14].

After reviewing all treatment alternatives, considering the patient’s history of surgical failures and size of the fistula, palate closure using a tongue graft was chosen as the preferred option since it ensured blood supply to the fibrous mucosa. On the upper arch, the treatment plan consisted of extractions of the primary maxillary canines and replacement of the lateral maxillary incisors with the permanent canines. Maxillary expansion using a hyrax-type appliance was chosen to correct the V-shaped arch form, and orthodontic fixed appliances were associated to achieve proper dental alignment. On the lower arch, the first premolars were extracted, and orthodontic fixed appliances were placed in order to correct the severe mandibular dental crowding (10.5 mm) and deep bite.

### 2.4. Treatment Progress

**Figure 3:**
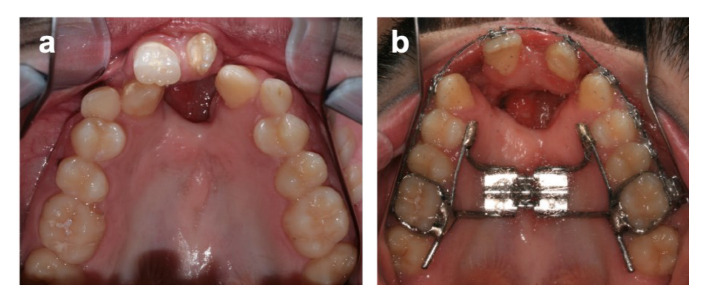
Upper arch before (**a**) and after (**b**) activation of hyrax-type appliance.

The primary maxillary canines were extracted, and a hyrax-type appliance cemented on the first molars and first pre-molars was placed. A semi-rapid expansion protocol was used (two activations per day) for 14 days, resulting in a 7.0 mm increase of the maxillary arch width on the first pre-molar area (Figure 3).

After maxillary expansion with the hyrax-type appliance, Roth 0.018 prescription fixed appliances were placed in order to level and align the arches using a normal archwire sequence. The severe mandibular dental crowding (10.5 mm) and the protrusion of mandibular anterior teeth were solved through extractions of the first mandibular pre-molars. The mandibular canine’s retraction was performed on a 0.016 × 22 stainless steel archwire using NiTi closing coils against the posterior groups, and this was followed by a careful retraction of the mandibular incisors so as to prevent fenestrations and gingival recessions since the patient had a thin gingival biotype.

**Figure 4:**
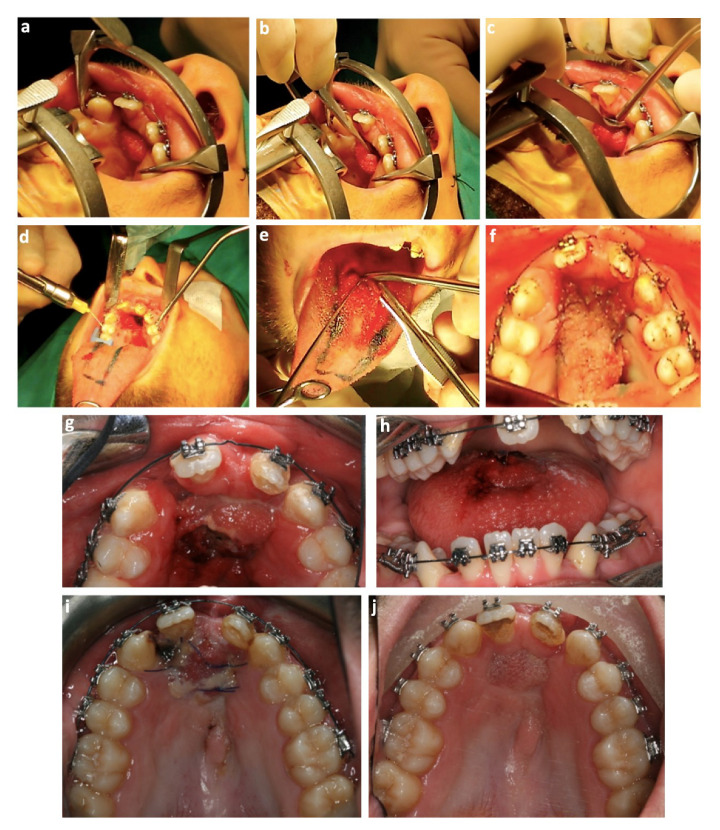
Intraoral photographs of the tongue flap: surgery (**a**) preparation of the operative field; (**b**) and (**c**) de-epithelized the fistulae edges; (**d**) anesthesia of the surgical tongue area; (**e**) tongue graft harvest; (**f**) tongue flap suture to the edges of the palatal defect; separation of the tongue pedicle (**g**,**h**); post-operatory photographs after 5 weeks (**i**) and 1 year (**j**).

The hyrax-type appliance was removed after retention for four months, and an anterior tongue flap surgery was performed (1 year after initiating orthodontic treatment). The surgery was performed when the patient was 19 years old, under general anesthesia and with nasotracheal intubation. First, the edges of the fistulae were de-epithelized to receive the flap, and subsequently, the flap was raised and rotated. At this point, suturing of the lower edge of the tongue was performed to assure hemostasis and close the space left by the anterior tongue flap. After that, the tongue was sutured to the edges of the palatal defect. For 28 days, the tongue graft was suspended between the cleft site and the tongue to maintain and guarantee vascularity at the cleft palate site. Then, a second surgery under local anesthesia was performed to detach the tongue pedicle and to reposition the donor site. In the following appointments, good healing was shown on both the flap and donor site without any signs of necrosis or relapse of the oronasal communication (Figure 4). After 6 months, a bone graft using cancellous bone from the iliac crest was performed in order to obtain bone support for teeth adjacent to the cleft.

After the graft surgery, in the maxillary arch a 0.016 × 0.022 stainless steel archwire was used to line up the maxillary dentition in a more mesial position by sliding mechanics with open-coil spring activation, replacing the absent lateral maxillary incisors for the canines. The mesialization of all posterior maxillary teeth was achieved by the use of elastic chains and Class III intermaxillary elastics when steel arch wires where place in both dental arches.

After the ideal positions of the anterior maxillary teeth were obtained, the mesial and distal spaces were maintained with a close coil spring in the inter bracket distance between teeth 13, 11, 21, and 23.

**Figure 5:**
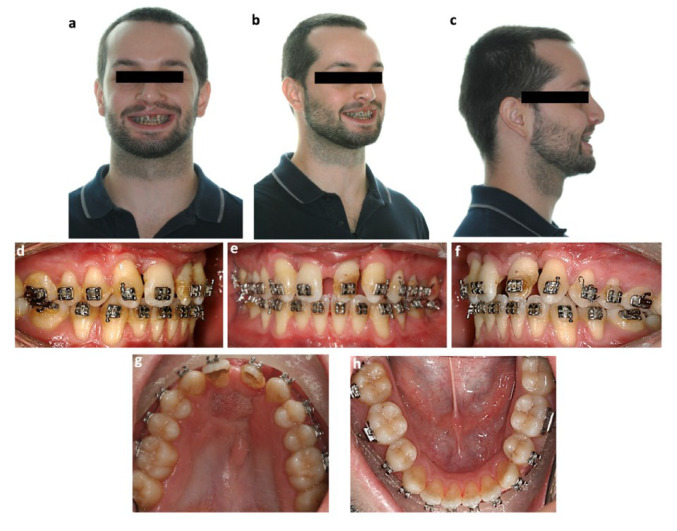
Post-orthodontic treatment photographs: extraoral (**a**) frontal view; (**b**) 3/4 lateral view; (**c**) profile and intraoral (**d**) right side; (**e**) frontal; (**f**) left side; (**g**) superior arch; (**h**) inferior arch.

During the finishing stage, for about 8 weeks, vertical triangular intermaxillary elastics were used to refine posterior dental intercuspation (Figure 5).

After 3 years and 6 months of active treatment, the fixed appliances were removed.

A diagnostic model was elaborated in which a diagnostic waxing was performed to close the interincisor diastema, change the coronary shape of teeth 11 and 21 with an increase in their mesiodistal dimension, and alter the coronary shape of teeth 13 and 23, transforming them into lateral incisors.

**Figure 6:**
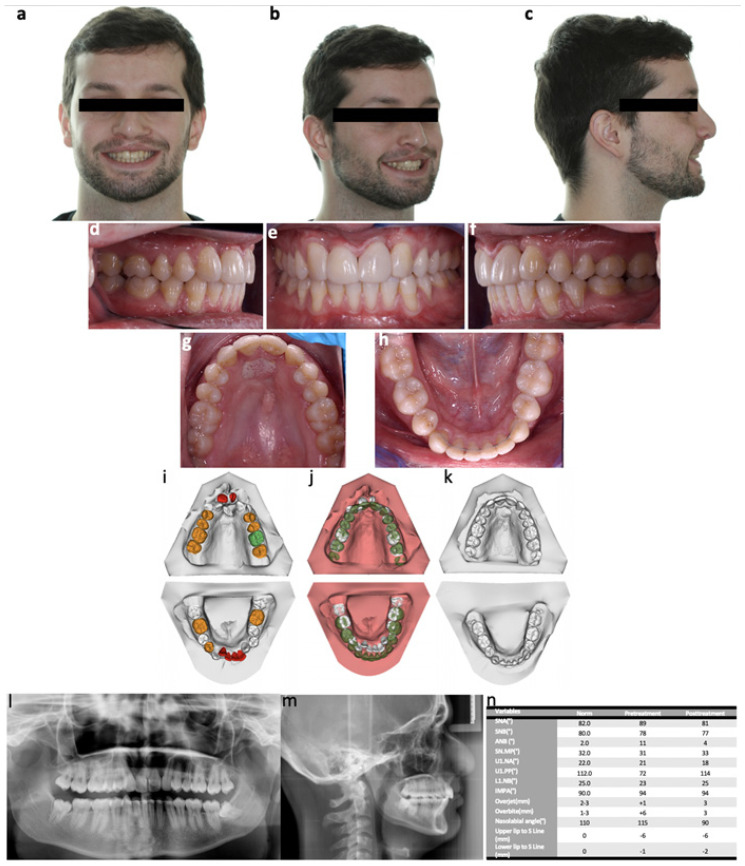
Post-treatment records: extraoral photographs (**a**) frontal view; (**b**) 3/4 lateral view; (**c**) profile; oral photographs (**d**) right side; (**e**) frontal; (**f**) left side; (**g**) superior arch; (**h**) inferior arch; dental casts (**i**) initial; (**j**) superimposition; (**k**) final; (**l**) final orthopantomography; (**m**) final lateral cephalogram; (**n**) cephalometric analysis.

The transfer of the diagnostic wax-up to the oral cavity was carried out using a soft silicone key. The planned restorations were performed after infiltrative anesthesia and with absolute isolation. Dental structure was conditioned with 37% orthophosphoric acid for 15 s and washed with air/water spray. The Optibond FL adhesive system (Kerr-Hawe, Brea, CA, USA) was applied over the conditioned enamel structure and photopolymerized for 20 s. The restoration’s stratification was performed using Filtek Supreme in layers of Dentin A2/A3, Body A2, Enamel A2, AT, and CT (3M-ESPE, St. Paul, MN, USA). Finishing and polishing of the resin restorations was carried out after 7 days using Diatech finishing drills (COLTENE WHALEDENT, Altstätten, Switzerland), So-flex Pop-On and SPIRAL SYSTEM discs (3M-ESPE, St. Paul, MN, USA), and interproximal strips (Figure 6).

In the mandibular arch, a lingual retainer was bonded to the 6 anterior teeth so as to maintain the intercanine width and ensure alignment stability. A Hawley plate retainer was made for the maxillary arch, and this appliance was used full-time for 6 months. After this period, it was used exclusively during the nighttime.

## 3. Results

At the end of the treatment, intraorally, the patient presented a bilateral Class I dental relationship. The maxillary midline appeared to be coincident with the facial midline, and the mandibular midline was slightly deviated to the left (0.5 mm). The retroinclination and overeruption of central incisors were correct, and a normal overbite and overjet were reached. The mandibular and maxillary arches were coordinated in an ovoid form. The changes in buccal corridors had a positive impact on smile esthetics, since the transverse widening of the maxillary arch was increased. Smile aesthetics was noticeably improved after the final restorations were performed, and the patient was greatly satisfied with the outcomes (Figure 6).

The post-treatment panoramic radiograph showed a good overall root parallelism, except for the left mandibular canine and right maxillary canine, which were tipped distally. The central incisors showed no increase in root resorption. The third maxillary molars erupted, but the mandibular third molars remained impacted.

The posttreatment cephalometric analysis showed a decrease of the ANB angle from 11° to 4° due to the retraction of the pre-maxilla. The inclination of the maxillary central incisors significantly improved from 72° to 114°. The mandibular incisor remained almost unchanged in relation to the mandibular plane angle (IMPA) and NB plane. The overbite was corrected, decreasing from 6.0 mm to 3.0 mm.

A slight rotation of the mandible increased the mandibular plane angle (as seen on superimposition tracings), and there was an increase in the lower anterior facial height. After the tongue graft, oral–nasal communication was closed, and no relapse was noticed.

The superimposition of models revealed that the dental arch morphology of both arches coincided, and the upper incisors inclination and lower incisors position and inclination has been corrected. According to this 3D analysis, the teeth that moved the most were the upper and lower incisors along with the 14 and 24.

Regarding facial aesthetics, the clinical team suggested that nasal tip correction be performed, but the patient refused to undergo another surgery as he was satisfied with his profile.

## 4. Discussion

The etiology of malocclusion in patients with CLP is attributed to several factors, which can be genetic and environmental. In this case, the clinical history excluded a genetic cause and identified prenatal environmental factors, such as the absence of prenatal medication with folic acid or excessive exposure to carbon monoxide, as the main predisposing risk factors.

Patients with bilateral CLP are more likely to have abnormities in number, shape, and size of teeth as well as changes in the enamel structure and teeth formation/eruption timing [15]. These features combined, or not, with environmental factors can contribute to the development of malocclusion in these patients. Multidisciplinary treatment should start in the neonatal period through neonatal orthopedic procedures that align and bring together the maxillary and lip segments before surgery in order to repair lip and palate defects. Nevertheless, these surgeries can cause maxillary vertical and/or anteroposterior deficiencies which can result in a retrognathic and constricted maxilla (possibly causing a crossbite and/or a V-shaped maxillary arch) [2,16,17].

In the reported case, the patient presented a CLP, skeletal Class II agenesis of the upper laterals and oronasal fistula persistence. Due to failure of three previous local mucosa flap surgeries, the oronasal fistula had significant scar tissue around the edges [7,18]. Additionally, the patient’s maxillary arch was collapsed and V-shaped, which could have been the result of several maxillary surgeries. These dental and occlusal changes are in accordance with previous studies that reported lateral maxillary incisors as the most frequently missing teeth in the cleft area [15,19,20,21,22]. Replacing the absent lateral maxillary incisors with canines can be the chosen treatment option since pleasant results in terms of aesthetics can be achieved and there is a good change in acceptance from the patient [23,24,25].

The maxillary expansion was performed with a hyrax-jackscrew-type appliance. This device was chosen after considering the required amount of transverse expansion (7 mm on the anterior region), the presence of scar tissue around the edges of the fistula (which is a difficult factor to overcome with a quad-helix-type appliance), and previous studies showing that slow and rapid maxillary expansion in bilateral cleft patients have similar results. In this case, the appliance design was modified so as to include extensions to the second molars [26,27]. In the present clinical case, it was found that the transverse distance obtained after the maxillary expansion remained stable without negative effects, such as dental tipping. These results are in agreement with a systematic review that reported that both tooth-borne and bone-borne devices had the same results in maxillary expansion, dental tipping, stability, and perceived pain [28]. However, a recent systematic review compared the effectiveness of different palatal expander approaches and concluded that, in the short term, skeletal expansion performed transverse expansion with fewer secondary effects, namely dental tipping. However, a long-term analysis revealed little supporting evidence [29].

After slow maxillary expansion, the fistula increased, as expected, from 12.0 mm × 13 mm to 33.0 mm × 14.0 mm (transversal × anteroposterior dimensions). This decision was reached after careful deliberation from Plastic Surgery, Maxillo-Facial Surgery, and Orthodontics departments. The expansion was carried out in order to correct the transverse discrepancy, establish the maxillary arch form, make a room for a bone graft by widening the alveolar cleft, and improve access to the alveolar bone graft area. Before secondary bone graft surgery, a tongue flap was used for oronasal fistula closure. This was due to several factors, which included the patient’s history of three failed soft tissue graft surgeries for palate closure between the ages 10 and 14, the presence of excessive scar tissue around the fistulae edges, and the increased dimensions of the fistula after the maxillary expansion. The osteogenic distraction protocol is an alternative treatment in these scenarios. However, in this case, it was not chosen since it requires several surgical reinterventions. Previous studies reported that when large fistula are present (wider than 10 mm), a pedicle tongue flap is the most successful surgical option for treatment of oronasal communication [9,18]. Moreover, in cases with excessive scarring or in which the cleft palate could be closed though previous palatal or vestibular flap surgeries, a tongue flap procedure can be the solution for a persisting oronasal fistulae [18,30].

There are two types of pedicle tongue flaps: anteriorly and posteriorly based. The posterior-based flaps are recommended in cases of soft palate, posterior mucosa, and retromolar area defects, while anterior-based flaps are indicated in cases of hard palate, anterior floor, oral mucosa, mouth, and lip defects [18]. Taking these indications into account, this patient was subjected to an anterior-based flap. To avoid tension during the healing process, the length and width of the tongue flap was 20% greater than the communication and had between five and seven millimeters of thickness in order to avoid any injuries to the vascular plexus [18,31]. After the tongue flap separation surgery, several controls were performed to assess healing. One year after surgery, there were no signs of necrosis or oronasal communication, which resulted in a significant improvement to the patient’s quality of life.

The orthodontic treatment goals were ultimately reached. The absence of the lateral incisor was solved with orthodontic movement for space closure. This treatment option was possible due to two factors: (1) the secondary bone graft surgery performed after the expansion protocol allowed for a satisfactory support of alveolar bone in both buccolingual and vertical directions; (2) the posterior teeth roots were in adequate condition. In this clinical case, due to resorting to this method, implant and prosthesis rehabilitation procedures were not necessary. Overall, adequate aesthetics and function were achieved and remained stable in the retention period.

## 5. Conclusions

In cases with severe changes in the dental and alveolar shape, treatment success can only occur with careful planning carried out by a multidisciplinary team with patient collaboration and individualized orthodontic forces to be applied. The tongue has proven to be an effective source for tissue transplantation, with a rich supply of blood. This procedure can be an alternative method to local mucosal flap surgeries, especially in patients with larger cleft defects and extensive fibrous areas.

## Data Availability

Not applicable.

## References

[1] Sabbagh H.J., Hassan M.H.A., Innes N.P.T., Elkodary H.M., Little J., Mossey P.A. (2015). Passive Smoking in the Etiology of Non-Syndromic Orofacial Clefts: A Systematic Review and Meta-Analysis. PLoS ONE.

[2] Sabri R., AbouJaoude N. (2021). Interdisciplinary management of a patient with a bilateral cleft lip and palate and 12 congenitally missing teeth. Am. J. Orthod. Dentofac. Orthop..

[3] Vyas T., Gupta P., Kumar S., Gupta R., Gupta T., Singh H. (2020). Cleft of lip and palate: A review. J. Fam. Med. Prim. Care.

[4] Pruzansky S. (1973). Cleft Lip and Palate: Therapy and Prevention. J. Am. Dent. Assoc..

[5] Gupta N., Shetty S., Degala S. (2020). Tongue flap: A “workhorse flap” in repair of recurrent palatal fistulae. Oral Maxillofac. Surg..

[6] Vasishta S.M.S., Krishnan G., Rai Y.S., Desai A. (2012). The Versatility of the Tongue Flap in the Closure of Palatal Fistula. Craniomaxillofac. Trauma Reconstr..

[7] Durmus Kocaaslan F.N., Tuncer F.B., Sendur S., Celebiler O. (2020). The tongue flap for large palatal fistulas, a success or a failure? Our 15-year experience. J. Plast. Surg. Hand Surg..

[8] Kim J.J., Alapati S., Knoernschild K.L., Jeong Y.-H., Kim D.G., Lee D.J. (2017). Micro-Computed Tomography of Tooth Volume Changes Following Post Removal. J. Prosthodont..

[9] Strujak G., Nascimento T.C.d.L.d., Biron C., Romanowski M., de Lima A.A.S., Carlini J.L. (2016). Pedicle Tongue Flap for Palatal Fistula Closure. J. Craniofac. Surg..

[10] Alkan A., Baş B., Özer M., Bayram M. (2007). Closure of a Large Palatal Fistula with Maxillary Segmental Distraction Osteogenesis in a Cleft Palate Patient. Cleft Palate-Craniofacial J..

[11] Zemann W., Pichelmayer M. (2011). Maxillary segmental distraction in children with unilateral clefts of lip, palate, and alveolus. Oral Surg. Oral Med. Oral Pathol. Oral Radiol. Endodontol..

[12] Zhang J., Zhang W., Shen S.G. (2018). Segmental Maxillary Distraction Osteogenesis With a Hybrid-Type Distractor in the Management of Wide Alveolar Cleft. Cleft Palate-Craniofacial J..

[13] Shahab N., Sar C., Sarac M., Erverdi N. (2019). Reconstruction of Premaxilla With Alveolar Distraction Osteogenesis in a Patient With Complete Cleft Lip and Palate: A Case Report. Cleft Palate-Craniofacial J..

[14] Mitsugi M., Ito O., Alcalde R.E. (2005). Maxillary bone transportation in alveolar cleft—transport distraction osteogenesis for treatment of alveolar cleft repair. Br. J. Plast. Surg..

[15] Ranta R. (1986). A review of tooth formation in children with cleft lip/palate. Am. J. Orthod. Dentofac. Orthop..

[16] Akarsu-Guven B., Karakaya J., Ozgur F., Aksu M. (2015). Growth-related changes of skeletal and upper-airway features in bilateral cleft lip and palate patients. Am. J. Orthod. Dentofac. Orthop..

[17] Vig K.W.L., Mercado A.M. (2015). Overview of orthodontic care for children with cleft lip and palate, 1915–2015. Am. J. Orthod. Dentofac. Orthop..

[18] Partida A.I.G., Lugo R.R. (2016). Reconstrucción de fístula palatina anterior con colgajo lingual de base anterior. Reporte de un caso. Rev. Odontológica Mex..

[19] Shaw W.C. (1979). Orthodontic Treatment of Malocclusion Associated with Repaired Complete Clefts of the Lip and Palate. Br. J. Orthod..

[20] Long R.E., Semb G., Shaw W.C. (2000). Orthodontic Treatment of the Patient with Complete Clefts of Lip, Alveolus, and Palate: Lessons of the past 60 Years. Cleft Palate-Craniofacial J..

[21] Šnmahel Z. (1994). Treatment Effects on Facial Development in Patients with Unilateral Cleft Lip and Palate. Cleft Palate-Craniofacial J..

[22] Olin W.H. (1964). Dental Anomalies In Cleft Lip And Palate Patients. Angle Orthod..

[23] Robertsson S. (2000). The congenitally missing upper lateral incisor. A retrospective study of orthodontic space closure versus restorative treatment. Eur. J. Orthod..

[24] Schneider U., Moser L., Fornasetti M., Piattella M., Siciliani G. (2016). Esthetic evaluation of implants vs canine substitution in patients with congenitally missing maxillary lateral incisors: Are there any new insights?. Am. J. Orthod. Dentofac. Orthop..

[25] Silveira G.S., de Almeida N.V., Pereira D.M.T., Mattos C.T., Mucha J.N. (2016). Prosthetic replacement vs space closure for maxillary lateral incisor agenesis: A systematic review. Am. J. Orthod. Dentofac. Orthop..

[26] Vasant M., Menon S., Kannan S. (2009). Maxillary Expansion in Cleft Lip and Palate using Quad Helix and Rapid Palatal Expansion Screw. Med. J. Armed Forces India.

[27] de Almeida A.M., Ozawa T.O., Alves A.C.d.M., Janson G., Lauris J.R.P., Ioshida M.S.Y., Garib D.G. (2017). Slow versus rapid maxillary expansion in bilateral cleft lip and palate: A CBCT randomized clinical trial. Clin. Oral Investig..

[28] Khosravi M., Ugolini A., Miresmaeili A., Mirzaei H., Shahidi-Zandi V., Soheilifar S., Karami M., Mahmoudzadeh M. (2019). Tooth-borne versus bone-borne rapid maxillary expansion for transverse maxillary deficiency: A systematic review. Int. Orthod..

[29] Coloccia G., Inchingolo A.D., Inchingolo A.M., Malcangi G., Montenegro V., Patano A., Marinelli G., Laudadio C., Limongelli L., Di Venere D. (2021). Effectiveness of Dental and Maxillary Transverse Changes in Tooth-Borne, Bone-Borne, and Hybrid Palatal Expansion through Cone-Beam Tomography: A Systematic Review of the Literature. Medicina.

[30] Yang Z., Zhong J., Chen W. (2020). Reconstruction of Large Anterior Palatal Fistulae Using Anteriorly Based Dorsal Tongue Flaps. J. Craniofac. Surg..

[31] Lahiri A., Richard B. (2007). Superiorly Based Facial Artery Musculomucosal Flap for Large Anterior Palatal Fistulae in Clefts. Cleft Palate-Craniofacial J..

